# The Clinical Presentations of Patients With COVID‐19 in the Omicron Era: Missed Opportunities in the Antiviral Treatment of SARS‐CoV‐2 Infections

**DOI:** 10.1002/jmv.70652

**Published:** 2025-10-21

**Authors:** G. Bartolucci, E. Garro, M. Mussa, A. Pepe, R. Angilletta, B. Rizzello, F. A. Ranzani, S. Bonora, G. Di Perri, A. Calcagno

**Affiliations:** ^1^ Department of Medical Sciences University of Turin Turin Italy; ^2^ Local Health Authority (ASL) Città di Torino Turin Italy; ^3^ Unit of Infectious Diseases, Department of Translational Medicine University of Western Piedmont Novara Italy; ^4^ Unit of Infectious Diseases Ospedale Maggiore della Carità Novara Italy

## Abstract

COVID‐19 has posed a significant global health challenge. SARS‐CoV‐2 has accumulated several mutations and adapted to humans, with its clinical manifestations evolving from severe bilateral interstitial pneumonia with respiratory failure to predominantly milder forms of disease. Effective antiviral treatments have been developed but remain underutilized, even in high‐risk individuals. We conducted a retrospective study on patients hospitalized for COVID‐19 in our hospital's Infectious Disease/COVID‐19 wards. Written informed consent was obtained from all participants. Demographic, clinical, and therapeutic data were collected. Due to limited viral sequencing, time periods were categorized based on the dominant epidemiological variants: Wild‐Type/Alpha (February 2020 to June 2021), Delta (July to December 2021), and Omicron (January 2022 to January 2024). We included 606 patients, of whom 381 (62.9%) were male, with a mean age of 65.5 years (±17.0) and a mean BMI of 26.4 kg/m² (±5.7). The most common comorbidities were arterial hypertension (43.8%), cardiovascular diseases (25.2%) and diabetes (21%). A total of 380 (62.7%)of patients were infected with the Wild‐Type variant, 49 (8.1%) with Wild‐Type/Alpha, 17 (2.8%)with Alpha, 20 (3.3%)with Alpha/Delta,45 (7.4%)with Delta, 4 (0.7%)with Delta/Omicron, and 91 (15%) with Omicron. The clinical presentation evolved significantly: we observed a decline in the prevalence of fever (78% in Wild‐Type vs. 57% in Omicron), myalgia (19% vs. 4%), diarrhoea (15% vs. 3%), rhinorrhoea (9% vs. 1%), and ageusia, which disappeared entirely in Omicron cases. In contrast, neurological symptoms became more frequent, increasing from 13% in Wild‐Type to 27% in Omicron infections, with delirium being the most common manifestation (59% of neurological cases). In‐hospital mortality was significantly lower with Omicron variants (5.5%) as compared to the older ones (Delta 15.9%, WT/alpha 13.2%, *p* < 0.001). Out of 256 participants with risk factors for severe disease, 116 (45.3%) could have potentially been treated with anti‐SARS‐CoV‐2 drugs for early treatment: only two patients (1.7%) were treated with such drugs in out‐of‐hospital settings. These results underscore the importance of targeted vaccination campaigns, enhanced access to early antiviral therapies, and further research to understand evolving clinical patterns and optimize therapeutic strategies.

## 1 Introduction

The SARS‐CoV‐2 virus, responsible for COVID‐19, has undergone several mutations over the course of its 4‐year existence [1]. Viral variants have emerged and disappeared, with some showing traits of increased contagiousness, immune evasion, or both. Since 2021, Omicron and its sub‐lineages have been the predominant SARS‐CoV‐2 variants. Its emergence marked a turning point in the clinical course of the infection: the reduced ability of this variant to infect the lower airways made it less virulent [2]. However, partial immune escape and shorter incubation periods have increased its contagiousness, leading to millions of cases annually [1, 2, 3, 4].

Data on hospitalization and mortality risks have significantly decreased from the early pandemic waves to the present. A study conducted in England reported a mortality rate of 0.67 per 1,000 people per year during the fifth wave [5], while the hospitalization rate dropped to 1.9% with the onset of Omicron [6]. The main risk factors include: age over 65, chronic lung disease, active cancer, cerebrovascular disease, kidney disease, chronic liver disease, diabetes mellitus, heart disease, severe obesity, immunodeficiency diseases or treatments, cystic fibrosis, disability, and psychiatric conditions [7].

Symptoms of Omicron infection are primarily rhinorrhea or nasal congestion (75%), headache (70%–75%), pharyngodynia (70%), and cough (40‐50%) [6, 8, 9]. Although vaccination has become less effective in preventing symptomatic disease since the emergence of Omicron, it remains highly effective in reducing the risk of hospitalization and death, even 180 days after administration of the current bivalent vaccines. The protection against symptomatic disease between 120 and 179 days is 24% [1, 10, 11].

NICE guidelines recommend nirmatrelvir‐ritonavir for patients with risk factors for severe COVID‐19 who do not require oxygen therapy [12]. Studies on the Omicron variant show this therapy reduces the relative risk of hospitalization by 69%, the risk of long‐COVID by 25%, and has a Number Needed to Treat (NNT) of 28 in high‐risk patients. Alternatively, although more suitable for hospitalised patients with respiratory failure, remdesivir remains effective against the Omicron variant even in outpatient settings. Monoclonal antibodies, on the other hand, represented a therapeutic option, but with the advent of new variants, particularly Omicron, they have lost their effectiveness and are no longer recommended [12, 13, 14, 15, 16].

## 2 Materials and Methods

This retrospective study, conducted at the University Clinic of Infectious Diseases of the ASL of Turin, examined the clinical characteristics of patients with COVID‐19 admitted from 21 February 2020 to 17 January 2024. The main objective was to investigate changes in the clinical picture of patients in relation to the emergence of new viral variants, the acquisition of collective immunity, the evolution of drug therapies and vaccination. The study was approved by the local Ethics Committee (Approval number 304/2020).

Data were collected from the discharge letters and the GALILEO management software. The discharge letters provided details on medical history, clinical course, procedures, therapies and complications, while the management software provided emergency room reports, laboratory analyses, radiological reports, specialist consultations and vaccination certificates.

Sex, age, BMI, risk factors, comorbidities and home drug therapy were recorded for each patient. The potential use of antiviral drugs was considered from March 2021 to January 2022 (monoclonal antibodies, 10 days from symptoms onset) and from January 2022 (5 days from symptoms onset) according to the availability of monoclonal antibodies or oral/intravenous antiviral drugs fro early treatment.

The date and symptoms experienced by the patients, the date of detection of the positive test, and admission were documented. Symptoms assessed included fever, pharyngodynia, rhinorrhoea, cough, dyspnoea, chest pain, lymphadenopathy, diarrhea, nausea/vomiting, myalgia, arthralgia, hyposmia, ageusia, asthenia, neuropathy, rash, bleeding, skin ulcers, conjunctivitis, and neurological symptoms, subdivided into headache, delirium, dizziness, syncope, and others.

The study analyzed the impact of different variants of SARS‐CoV‐2 on the clinical severity of hospitalized patients, using data from the Istituto Superiore di Sanità (ISS) flash surveys to determine which variant was responsible for the infection. The wild‐type variant maintained prevalence until 18 February 2021, followed by the Alpha variant until 22 June 2021, the Delta variant until 16 December 2021, and finally the Omicron variant, which is still dominant. For the data analysis, the wild‐type and Alpha variants were considered as a single variant [17, 18, 19, 20, 21, 22, 23, 24, 25, 26, 27, 28, 29, 30, 31, 32, 33, 34, 35, 36, 37, 38, 39, 40, 41, 42, 43, 44].

Data are shown as mean (standard deviation) or number (percentage). Parametric tests were used for statistical analysis through the software SPSS (vers. 18.0).

## 3 Results

We included 606 participants, of whom 381 (62.9%) were male, with a mean age of 65.5 years (±17.0); 326 participants (53.8%) were older than 65 years. Baseline features are shown in Table 1. The mean BMI was 26.4 kg/m² (± 5.7). The most common chronic diseases were: arterial hypertension cardiovascular diseases, diabetes, chronic neurological diseases, and chronic obstructive pulmonary diseases. Table 1 summarizes the demographic and clinical features of study participants according to SARS‐CoV‐2 variant period. 75(12,4%) participants died during hospitalization.

**Table 1:** Baseline characteristics and medical history of participants stratified by SARS‐CoV‐2 viral variant.

	All	Wild‐type/Alpha	Delta	Omicron	*p* value
*n*	606	446	69	91	—
Age (years)	65.5 (17.0)	65.1 (16.9)	56.0 (16.3)	74.5 (13.5)	< 0.001
Male sex at birth	381 (62.9%)	288 (64.6%)	45 (65.2%)	48 (52.7%)	0.103
Body mass index (Kg/m^2^)	26.4 (5.7%)	26.3 (5.4%)	27.7 (8.7%)	26.2 (5.2%)	0.667
Smoking habit:					
Previously	83 (17.9%)	56 (18%)	10 (16.4%)	17 (18.7%)	0.368
Actively	27 (5.9%)	16 (5.2%)	3 (5%)	8 (8.8%)	
Hazardous alcohol consumption	4 (0.8%)	4 (1.2%)	0 (0%)	0 (0%)	0.392
Number of medications taken	3.2 (3.5%)	2.9 (3.2%)	2.4 (3.5%)	5.1 (3.8%)	< 0.001
Arterial hypertension	242 (43.8%)	168 (42.9%)	24 (34.8%)	50 (54.9%)	0.030
Cardiovascular disease	139 (25.2%)	93 (23.7%)	14 (20.3%)	32 (35.2%)	0.050
Diabetes	116 (21.0%)	76 (19.4%)	12 (17.4%)	28 (30.8%)	0.035
Neurological disease	68 (14.2%)	36 (11.3%)	2 (2.9%)	30 (33%)	< 0.001
COPD	56 (10.2%)	34 (8.7%)	2 (2.9%)	20 (22%)	< 0.001
Active cancer	42 (7.6%)	27 (6.9%)	3 (4.3%)	12 (13.2%)	0.072
Chronic kidney failure	31 (5.6%)	20 (5.1%)	2 (2.9%)	9 (9.9%)	0.120
Asthma	25 (5.3%)	18 (5.7%)	3 (4.3%)	4 (4.4%)	0.822
Tubercolosis	15 (3.2%)	10 (3.2%)	1 (1.5%)	4 (4.4%)	0.582
HIV	13 (2.4%)	9 (2.4%)	2 (2.9%)	2 (2.2%)	0.923
HBV	11 (2.0%)	9 (2.4%)	0 (0%)	7 (7.7%)	< 0.001
HCV	9 (1.7%)	6 (1.6%)	1 (1.5%)	2 (2.2%)	0.914
Liver cirrhosis	7 (1.3%)	5 (1.3%)	1 (1.5%)	1 (1.1%)	0.978
Solid organ transplantation	4 (0.7%)	3 (0.8%)	1 (1.4%)	0 (0%)	0.557
Asplenia	3 (0.6%)	3 (0.9%)	0 (0%)	0 (0%)	0.468

**Figure 1:**
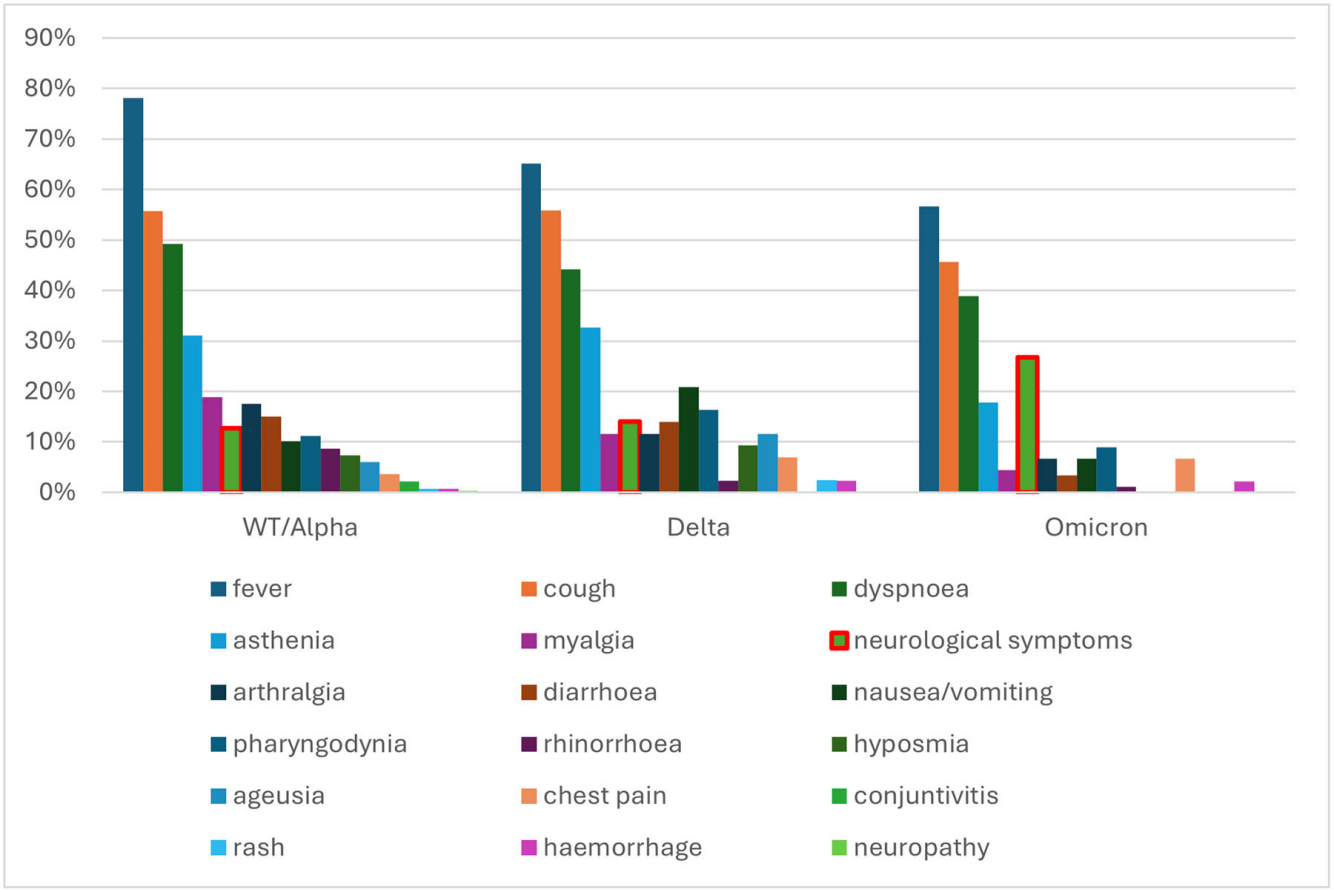
Distribution of reported symptoms according to SARS‐CoV‐2 viral variants.

While 16(2,9%) study participants were asymptomatic, the most commonly reported symptoms were fever395 (72.3%)participants, followed by cough 286 (52.7%)participants, dyspnoea (45.6%, 249 participants) and asthenia (27%, 135 participants). Among neurological symptoms, headache was the most frequent (40.3%, 31 participants), followed by delirium (35.5%, 27 participants), syncope (10.5%, 8 participants), and dizziness (7.9%, 6 participants). The prevalence of symptoms at diagnosis is shown in Figure 1.

Of the 217 patients eligible for anti‐COVID‐19 vaccination, 90 participants(41.5%) had received at least one dose. The vaccination regimen was: one dose 12 participants (14.6%), two doses 14 participants (15.7%), three doses 30 participants (33.7%), four doses 26 participants (29.2%) and five doses (6 participants 6.7%).

### 3.1 Differences According to Viral Variants

The study identified 380 patients (62.7%) infected with the Wild‐type variant, 49 (8.1%) with Wild‐type‐Alpha, 17 (2.8%) with Alpha, 20 (3.3%) with Alpha‐Delta, 45 (7.4%) with Delta, 4 (0.7%) with Delta‐Omicron and 91 (15%) with Omicron.

The analysis of symptoms for the different variants showed that fever was the most common symptom, with a prevalence ranging from 56.7% (Omicron) to 78.1% (Wild‐type). Cough and dyspnoea were frequent in all variants, while asthenia ranged from 32.6% (Delta) to 17.6% (Alpha). Neurological symptoms ranged from 26.7% (Omicron) to 12.7% (Wild‐type). Symptoms such as ageusia and hyposmia disappeared with Omicron, whereas conjunctivitis, present with Wild‐type, is not documented in the other variants. Pharyngodynia and rhinorrhoea were less common but present to varying degrees. Gastrointestinal symptoms such as diarrhoea and nausea/vomiting were reported, with Delta showing the highest percentage of nausea/vomiting (20.9%).

The chi‐square test indicated statistically significant differences between the variants for fever, rhinorrhoea, diarrhoea, myalgias, ageusia, rash and neurological symptoms

The data of the investigated and most relevant symptoms are summarised in Table 2.

**Table 2:** Reported symptoms according to SARS‐CoV‐2 viral variant.

Symptom	All	Wild‐type	Alpha	Delta	Omicron	*p* value
Fever	395 (72.3%)	250 (78.1%)	12 (70.6%)	28 (65.10%)	51 (56.70%)	< 0.001
Cough	286 (52.7%)	177 (55.7%)	9 (52.9%)	24 (55.80%)	41 (45.60%)	0.239
Dyspnoea	249 (45.6%)	157 (49.2%)	7 (41.2%)	19 (44.20%)	35 (38.90%)	0.389
Asthenia	135 (27.0%)	85 (31.1%)	3 (17.6%)	14 (32.60%)	16 (17.80%)	0.115
Myalgias	83 (15.2%)	60 (18.9%)	3 (17.6%)	5 (11.60%)	4 (4.40%)	0.027
Neurological symptom	71 (13.9%)	36 (12.7%)	0 (0%)	6 (14%)	24 (26.70%)	0.003
Arthralgia	67 (13.4%)	48 (17.5%)	1 (5.9%)	5 (11.60%)	6 (6.70%)	0.125
Diarrhoea	67 (12.3%)	48 (15.0%)	3 (17.6%)	6 (14%)	3 (3.30%)	0.046
Nausea/vomiting	52 (10.3%)	28 (10.1%)	1 (5.9%)	9 (20.90%)	6 (6.70%)	0.166
Pharyngodinia	46 (9.3%)	30 (11.2%)	0 (0%)	7 (16.30%)	8 (8.90%)	0.038
Rhinorrhoea	29 (5.9%)	23 (8.6%)	0 (0%)	1 (2.30%)	1 (1.10%)	
Hyposmia	32 (5.9%)	23 (7.3%)	2 (11,7%)	4 (9.30%)	0 (0%)	0.061
Ageusia	30 (5.5%)	19 (6%)	17 (100%)	5 (11.60%)	0 (0%)	0.007
Thoracic pain	21 (4.2%)	10 (3.6%)	1 (5.90%)	3 (7%)	6 (6.70%)	0.580
Congiuntivitis	7 (1.4%)	6 (2.2%)	0 (0%)	0 (0%)	0 (0%)	0.699
Rash	5 (1.0%)	2 (0.7%)	1 (5.90%)	1 (2.40%)	0 (0%)	< 0.001
Emorrhagia	5 (1.0%)	2 (0.7%)	0 (0%)	1 (2.30%)	2 (2.20%)	0.783
Neuropathy	3 (0.5%)	1 (0.3%)	1 (5.90%)	0 (0%)	0 (0%)	0.061

Among the 77 patients who experienced neurological symptoms, headache was the most common symptom, followed by delirium, syncope and dizziness. To investigate the association between these symptoms and the SARS‐CoV‐2 viral variants, the grouped variants wild‐type/Alpha, Delta and Omicron were considered.

**Figure 2:**
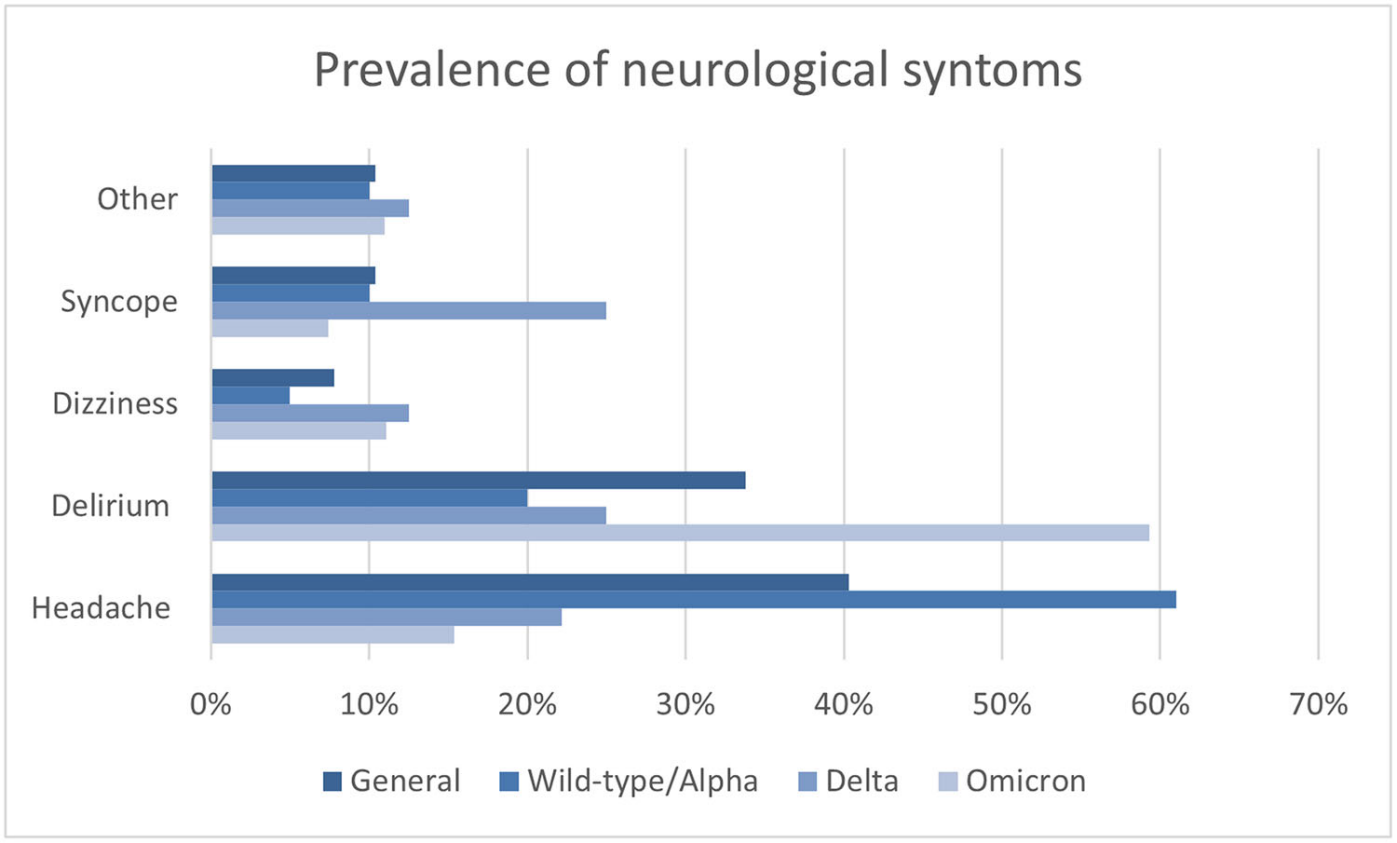
Prevalence of neurological symptoms across different SARS‐CoV‐2 viral variants.

The pooled data show that headache is the predominant symptom in the wild‐type/Alpha variant, whereas delirium is the predominant symptom in the Omicron variant. In contrast, data on syncope and dizziness did not reach statistical significance. Data on nerurological symptoms are summarised in Figure 2 and Table 3.

In‐hospital mortality was significantly lower with Omicron variants (5.5%) as compared to the older ones (Delta 15.9%, WT/alpha 13.2%, *p* < 0.001).

Among the study participants, 42 patients had active malignancies. Of these, only 10 received early antiviral therapy: 8 were treated with remdesivir during hospitalization, 1 with nirmatrelvir/ritonavir, and 1 with molnupiravir. Among those who received remdesivir, two patients died during their hospital stay.

**Table 3:** Neurological symptoms observed by SARS‐CoV‐2 viral variant.

Symptom	All	Wild‐type/Alpha	Delta	Omicron	*p* value
Headache	31 (40.3%)	25 (61.0%)	2 (22.2%)	4(15.4%)	*p* < 0.001
Delirium	26 (22.8%)	8 (20.0%)	2 (25%)	16 (59.3%)	*p* = 0.003
Dizziness	6 (7.8%)	2 (5.0%)	1 (12.5%)	3 (11.1%)	*p* = 0.587
Syncope	8 (10.4%)	4 (10.0%)	2 (25.0)%	2 (7.4%)	*p* = 0.360
Other	8 (10.4%)	4 (10.0%)	1 (12.5%)	3 (11.0%)	*p* = 0.974

### 3.2 Antiviral Treatment

Out of 256 participants with risk factors for severe disease, 116 (45.3%) could have potentially been treated with anti SARS‐CoV‐2 drugs for early treatment (within 10 or 5 days from symtoms onset and with available monoclonal antibodies or antiviral drugs). Out of 116 only only two patients (1.7%) had started therapy in out‐of‐hospital settings. The first case was aa 83‐year‐old man with a BMI of 31, a history of smoking, and active neoplasia. tixagevimab/cilgavimab was administered late, on the ninth day after the onset of symptoms: the patient did not receive the second dose because he manifested significant desaturation, necessitating hospitalization on the same day. The second case was a 42‐year‐old woman, with active neoplasia and asymptomatic at the time of the positive test for SARS‐CoV‐2. The patient started treatment with nirmatrelvir/ritonavir, but after only 1 day, a picture of leukopenia (950 10^6/l, with 140 10^6/l lymphocytes) was revealed, so hospitalization was deemed appropriate.

## 4 Discussion

The clinical presentation of COVID‐19 changed significantly when massive vaccination programs were completed, and viral variants were selected and became dominant. Understanding COVID‐19 pathomorphosis is essential for a prompt diagnosis and early antiviral treatment in high‐risk individuals.

The most frequently observed symptoms in patients were fever, cough, and dyspnoea, followed by asthenia, myalgia, and neurological symptoms. The presentation differed slightly from the literature, with pharyngodynia, headache, gastrointestinal symptoms, ageusia, and conjunctivitis less frequent [45]. This could indicate that the virus, in addition to replicating in the upper airways, directly reaches the alveoli causing acute inflammation and more severe disease. However, the selection bias of our study (hospitalized patients only) could explain these findings.

Analysing the variants, we observed that fever was less common with the evolution of the virus: in the Wild‐Type variant, fever was found in 78% of patients, while in the Omicron variant in 57%. Myalgias also showed a similar trend: from 19% in Wild‐Type to 4% in Omicron. Diarrhoea decreased from 15% in Wild‐Type to 3% in Omicron; rhinorrhoea from 9% to 1%, while ageusia disappeared (from 6% to no cases recorded). Rash, less present with Wild‐Type and Omicron, occurred only in Alpha (6%) and Delta (2%) variants. Neurological symptoms are more common in the Omicron variant, ranging from 13% in Wild‐Type to 27% in Omicron. These data are similar in the literature, albeit in different proportions, especially for the Omicron variant: rhinorrhoea (75% vs. 1%), myalgias (30% vs. 4%), diarrhea (15% vs. 3%) and ageusia (3% vs. 0%) are less frequent, while fever (35% vs. 57%) is more frequent [6, 8].

One of the most relevant findings of our study is the high prevalence of neurological symptoms and its increase in participants infected with Omicron variants. Delirium is the most frequent neurological symptom in the Omicron variant, found in 59% of patients with neurological symptoms, a higher percentage than in the other variants. Mental confusion is reported as a common symptom in the literature for the Omicron variant, occurring in 25%–30% of patients. The figure for delirium could be influenced by the advanced age of patients, who are generally more susceptible to this symptom in the presence of fever or hospitalization. However, the literature shows a higher frequency of delirium in patients with COVID‐19 than in other diseases [46]. This aspect should be investigated as delirium prolongs the duration of hospitalization and leads to sequelae in frail patients, as well as a possible passage of inflammatory cytokines to the CNS. Geriatric literature identifies delirium as a short and long‐term cause of mortality in older patients [47].

The second most important result of our study was the observation that the vast majority of our study participants did not receive early antiviral treatment at home. Only two patients started early antiviral therapy outside the hospital setting, corresponding to less than 1% of those who could have benefited from it. These data highlight challenges in primary care and the need to increase efforts to avoid hospitalization of patients, especially frail ones. The drug nirmatrelvir‐ritonavir, available in Italy from 27 February 2022, has not been used as recommended, even more than a year after its introduction in the market, as the numbers in our cohort show. Early antiviral therapy in omicron‐infected patients showed consistent benefits (in terms, of reduced mortality or hospitalization) in high‐risk individuals [14]. These missed opportunities are not only detrimental to patients, who could have avoided hospitalization but also increase the stress on healthcare facilities. Our study, however, was not able to fully analyze the impact of early treatment as only hospitalized patients were analyzed. However, if we apply the data from a Canadian study in patients infected with the omicron variant, early antiviral treatment with nirmatrelvir/ritonavir, we can estimate the number of potentially avoided hospitalizations. Considering a number needed to treat of 37 (24–50) and 309 high‐risk patients since 2022, 8 (6–12) hospitalizations could have been potentially avoided.

Our study was unable to analyse the reasons for the failure to prescribe early antiviral therapy in the patients examined. We had multiple discussions with general practitioners on COVID‐19 diagnosis and treatment, and several hypotheses for this issue have been hypothesized such as the low perception of COVID‐19 complications in recent years, the time‐wasting online procedure for obtaining AIFA's authorization and understaffing.

The main limitations of this study concern its single‐centre, retrospective design and the exclusive enrolment of hospitalised patients, which could reduce the generalisability of the results to the general population. A further limitation is the approximation in the identification of the viral variant, which could not be confirmed by specific laboratory tests.

Despite these advances, COVID‐19 still represents a challenge for public health. The efforts of physicians and researchers must continue to prevent the hospitalisation of frail participants, who remain the most vulnerable. An annual vaccination campaign, specifically targeting frail patients, is essential to maintain a high level of protection from hospitalisation. In addition, the early use of antiviral therapies can make a difference in limiting disease progression in at‐risk participants. Further research is essential to develop new therapeutic strategies and to better understand the mechanisms of immunity and resistance to the virus.

## Conflicts of Interest

The authors declare no conflicts of interest.

## Data Availability

The data that support the findings of this study are available from the corresponding author upon reasonable request.
